# Costs and cost-efficiency of a mobile cash transfer to prevent child undernutrition during the lean season in Burkina Faso: a mixed methods analysis from the MAM’Out randomized controlled trial

**DOI:** 10.1186/s12962-018-0096-9

**Published:** 2018-04-13

**Authors:** Chloe Puett, Cécile Salpéteur, Freddy Houngbe, Karen Martínez, Dieynaba S. N’Diaye, Audrey Tonguet-Papucci

**Affiliations:** 1 Research and Technical Department, Action Against Hunger, New York, NY USA; 2 Department of Expertise and Advocacy, Action contre la Faim, Paris, France

## Abstract

**Background:**

This study assessed the costs and cost-efficiency of a mobile cash transfer implemented in Tapoa Province, Burkina Faso in the MAM’Out randomized controlled trial from June 2013 to December 2014, using mixed methods and taking a societal perspective by including costs to implementing partners and beneficiary households.

**Methods:**

Data were collected via interviews with implementing staff from the humanitarian agency and the private partner delivering the mobile money, focus group discussions with beneficiaries, and review of accounting databases. Costs were analyzed by input category and activity-based cost centers. cost-efficiency was analyzed by cost-transfer ratios (CTR) and cost per beneficiary. Qualitative analysis was conducted to identify themes related to implementing electronic cash transfers, and barriers to efficient implementation.

**Results:**

The CTR was 0.82 from a societal perspective, within the same range as other humanitarian transfer programs; however the intervention did not achieve the same degree of cost-efficiency as other mobile transfer programs specifically. Challenges in coordination between humanitarian and private partners resulted in long wait times for beneficiaries, particularly in the first year of implementation. Sensitivity analyses indicated a potential 6% reduction in CTR through reducing beneficiary wait time by one-half. Actors reported that coordination challenges improved during the project, therefore inefficiencies likely would be resolved, and cost-efficiency improved, as the program passed the pilot phase.

**Conclusions:**

Despite the time required to establish trusting relationships among actors, and to set up a network of cash points in remote areas, this analysis showed that mobile transfers hold promise as a cost-efficient method of delivering cash in this setting. Implementation by local government would likely reduce costs greatly compared to those found in this study context, and improve cost-efficiency especially by subsidizing expansion of mobile money network coverage and increasing cash distribution points in remote areas which are unprofitable for private partners.

## Background

Cash-based approaches in humanitarian emergencies have been used to address various conditions affecting vulnerable populations such as improvement of maternal health and adherence to immunization schedules [1]. Growing evidence shows that cash can be an effective measure to address undernutrition, although evidence differs across programs [2–4]. Compared to other interventions such as water and sanitation and food or voucher transfer, cash transfers enable affected populations to make choices about their own needs, can boost local markets, and require minimal staff and infrastructure for implementation especially if mobile phones are used [5]. Beyond the relative ease and efficiency of transferring cash via mobile phone networks [6], mobile transfers also ensure privacy of beneficiaries and enable stricter security measures [7].

Depending on the presence or absence of restrictions placed on the use of cash among recipients, transfers are defined as being conditional or unconditional. Unconditional cash transfers (UCT) commonly are used in humanitarian settings [7, 8].

The efficiency of cash transfers is usually measured by the cost-transfer ratio (CTR). The CTR represents all the costs required to transfer one monetary unit (in this case a dollar) to a beneficiary, excluding the cost of the transfer itself; the total cost transfer ratio (TCTR) is the total cost to transfer one monetary unit to a beneficiary, including the value of the transfer [9]. Assessment of CTRs also shows that cash transfers are generally (although not always) more efficient to deliver than either vouchers or in-kind transfers [6]. For example, a study in Ecuador found food rations to carry the highest marginal cost per transfer at 11.50 USD compared to approximately 3.00 USD per transfer for cash and vouchers. While vouchers were considered the most cost-effective option and the most effective in increasing dietary diversity, households reported a preference for cash since it allowed autonomy in food choice [10]. Similarly, in Niger, cash and food transfers were delivered at the same frequency, and costs for the food transfer were 15% higher [11].

While these previous findings indicate that cash holds potential as a relatively affordable food assistance option, this varies according to its implementation modality in a given context and the way in which it is implemented [6]. A gap in evidence remains regarding the efficiency of cash transfers. No satisfactory evaluation has been published on the cost needed to implement this intervention, especially one accounting for the costs borne by program beneficiaries [6]. Moreover, policy makers need evidence-based results to decide the most cost-efficient cash transfer option to implement in their respective context.

The MAM’Out study is a two-arm randomized control trial (RCT) implemented in Burkina Faso by Action Against Hunger (a humanitarian nongovernmental organization) and supported by four academic partners [12]. This trial compared the impact of a multiannual seasonal UCT on acute malnutrition incidence among young children compared to a control group receiving no intervention. While no impacts were found on the primary outcome of acute malnutrition incidence [13], a positive impact was demonstrated on child nutrient intake, and child and caregiver dietary diversity [14]. The cash transfers distributed as part of this trial were unconditional, and no restriction was imposed on the use of the cash. However, community sensitization sessions organized with participant caregivers in all villages highlighted the need to use the money to support their child’s development and to prevent undernutrition. This information was further recalled during visits to and discussions in communities by field staff. A qualitative study from the trial found the cash primarily was used for food and health care for the child and the family [15].

This paper documents an economic analysis conducted alongside the MAM’Out trial. The objective of this analysis was to assess cost and cost-efficiency of this intervention, taking a societal perspective by including costs to implementing partners and households.

## Methods

### Context

Burkina Faso is a landlocked country located in the Sahel region of West Africa. With more than 18 million inhabitants, it is ranked 185 out of 188 according to the 2015 Human Development Index [16]. The government jointly with technical and financial partners recently promoted social transfer mechanisms for the poorest and most vulnerable households to enhance food security [17].

The eastern region experiences a high prevalence of acute malnutrition, making the region a priority area for addressing undernutrition [18]. In Tapoa province, the prevalence of global acute malnutrition among children aged 6–59 months (2006 WHO growth reference) was estimated at 17.3% (95% CI 15.2–19.7) in April 2012, above the WHO critical threshold, demonstrating a need for intervention [19].

Action Against Hunger, hereafter referred to as the “humanitarian agency”, has been present in Tapoa province since 2008 and implements programs in water, sanitation, hygiene, food security and nutrition by providing financial and technical support to the national health system. In 2012, the results of a Nutrition Causal Analysis conducted by the humanitarian agency indicated that women’s financial insecurity was one of the major perceived causes of undernutrition [20]. Due to their multi-sectoral nature, cash transfers can act at different levels, safeguarding direct determinants of child undernutrition: child food intake, child care and child morbidity [13]. A feasibility study conducted in November 2012 provided details on operational guidance for a cash based intervention targeted to vulnerable households.

The MAM’Out research project aimed to assess the effectiveness of multiannual seasonal UCTs to prevent acute malnutrition among young children in rural Burkina Faso [12]. The project was implemented from June 2013 to October 2015 after acceptance from the ethics committee of Ghent University Hospital in Belgium and the Burkinabe National Ethics Committee. The research was also registered on clinicaltrial.gov (NCT01866124). The study methods have been described elsewhere [13]. The project was implemented in 32 villages in Kantchari commune, northern Tapoa province. Inclusion criteria were: households classified as poor or very poor according to the household economy approach [21] and having at least one child under 12 months of age at inclusion. Thirty-two villages were randomly assigned to either intervention (n = 16) or control group (n = 16). The intervention consisted of a seasonal UCT provided from July to November over 2 years, in 2013 and 2014. The distribution period overlapped with the annual rainy season from May to August, known as the “hunger” season, with decreased food availability and increased physical activities for agricultural work, and consequent increased nutritional needs.

The transfer size was estimated based on previous cash transfer experiences in Burkina Faso and in the sub-Sahara region [22]. Mothers were identified as recipients of the transfers since they are usually in charge of childcare. Each month, 10,000 CFA (Communauté financière d’Afrique, or West African franc) (≈ US$17) were transferred to mothers in participating households. Over a year, a total of 50,000 CFA (≈ US$85) was given to each household in the intervention group. The transfer size represented approximately 33% of Burkina Faso’s 2014 national poverty line (estimated at 153,530 CFA ≈ US$260) [23].

Cash was transferred using mobile phones in partnership with a mobile phone company operating in the country, referred to hereafter as the “private partner”. Transfer using mobile phone was chosen to maintain beneficiary privacy and for security reasons. A total of 893 mothers from 856 households were identified as primary recipients since they are usually in charge of child care. All of them received a mobile phone and a charger, a sim card linked to a mobile account and a MAM’Out project identity card at a preliminary session organized at each selected cash withdrawal point. Fixed or mobile cash withdrawal points were located on average at 5–10 km away from villages. Before the cash distribution, mothers received a text message with a code number notifying them that their account was credited. Mothers were thus invited to visit cash withdrawal points to collect their money. Presentation of the MAM’Out identity card and the code number granted access to the money. Mothers confirmed the withdrawal of cash by signing follow up lists.

### Data collection

Data collection tools were developed based on previous studies [24–26] to collect information on the following topics: staff time allocation (time allocation interviews with program staff at humanitarian agency and private partner); resources used in implementation (interviews with private partner program and accounting staff); direct and indirect costs related to program participation (focus group discussions (FGD) with program beneficiaries) and wage rates available for local livelihoods (focus group discussions with program beneficiaries). A field visit for cost data collection was undertaken in October and November 2014. Data was collected from different sources, as outlined in Table 1 and described below.

**Table 1:** Data sources used in analysis

Data source	# Interviews	# Participants (group discussions)	Topics covered
Accounting data	2^a^		Direct and indirect costs incurred by implementing institutions
Staff FGD	1	20	Program activities
Beneficiary FGDs	5	9 each (45 total)	Costs incurred by beneficiaries and locally-available wage rates
Key informant interviews	19	–	
Humanitarian agency staff	12	–	Time allocation to activities
Private partner staff	4	–	Time allocation to activities, other resources used, including security measures
Cash point staff	3	–	Time allocation to distributions, other resources used, including security measures

*FGD* focus group discussion

^a^Accounting staff at humanitarian agency and private partner

Interviews and FGDs with program staff were conducted by the primary researcher (CP) with the aid of a French–English translator. All accounting databases contributing funding to the Kantchari base office of the humanitarian agency were reviewed with the accountant and project staff, to identify budget lines contributing to the project. Private partner accounting staff provided summary budgetary information on the agency’s involvement in the cash transfers; additional data on resources used by partners in implementation were collected during interviews.

A FGD with all humanitarian agency field staff was conducted at the beginning of the data collection visit, and covered questions related to main activities in the program for the various staff profiles, and whether staff would categorize each activity as primarily for research or operations. The FGD was loosely structured around this primary question and was conducted to understand better the program activity structure and develop a comprehensive activity list for subsequent key informant interviews with staff on their activity time allocation. Following this group discussion, interviews were conducted with individual staff involved in the project, and questions were asked regarding their activities undertaken as part of the program and their time allocation to these activities. Nineteen key informant interviews were conducted with humanitarian agency and private partner staff in the capital, Ouagadougou, and in the field in Kantchari. Discussions were held with humanitarian agency staff (n = 12) involved in operations, research and support roles, and with private partner staff (n = 4) including managers and staff involved in overseeing the distribution. Further interviews were conducted with individual staff at two cash points (n = 3), including one bank and one community center where cash was distributed as part of the intervention. Where it was not feasible to conduct an interview with a staff person, discussions were held with their supervisors and colleagues to determine and triangulate their level of involvement in the program. Time allocation interviews were conducted at the end of the second year of implementation; the recall period for these interviews was the entire implementation period of 1.5 calendar years (April 2013 to October 2014).

FGDs were also conducted with program beneficiaries; these discussions were conducted by a trained facilitator in the local language of Gourmanchéma, handwritten notes were recorded by a note-taker, and sessions were audio recorded. Five FGDs were conducted with 9 beneficiaries each. Villages for FGDs were selected purposively to provide variation in the location and the type of distribution point attended, whether fixed or mobile. The purpose of this selection process was to capture a variety of beneficiary experiences in different geographic areas and attending different kinds of distribution points. Personal identifying data such as age and ethnicity were not included in the selection criteria. Questions were asked regarding time spent participating in the program, particularly time spent waiting, and out of pocket costs incurred in attending distributions and otherwise participating in the program. Questions were also asked about time spent participating in FGDs with the humanitarian agency during the program months. In-depth community discussions were chosen as the method for collecting participant cost data, rather than a quantitative survey, to understand better the wage rates available for local livelihoods, and to ensure beneficiaries understood questions related to their time spent in the various activities.

### Data analysis

This study used standard methods for economic evaluation within Action Against Hunger [27]. This included taking a societal approach to cost analysis whereby all costs involved in program implementation were collected, regardless of who incurred them. This approach includes both financial costs from organizational accounting systems and economic costs to beneficiary households and communities incurred during the time horizon of the MAM’Out trial.

### Cost estimation

Institutional accounting data was adjusted in several stages to calculate cost estimates.

Costs considered appropriate for inclusion in this analysis were for the operational component of MAM’Out. Both research and operations staff were asked about their time allocated to activities which could affect nutrition outcomes. The costs of research activities, and time spent on them, as identified during the staff FGD, were excluded from the analysis for two reasons: (1) because these activities are conducted to observe, and not to influence, nutrition outcomes, and (2) because excluding these costs produces cost estimates that would represent better the costs of a standard cash transfer program. This was done using staff time allocation interviews with key informants, who estimated time spent on research and operational activities. Higher-level research support costs provided from the humanitarian agency headquarters in France were excluded as they supported primarily the research component and not direct implementation. Costs for specific studies which did not influence program functioning (e.g. food recall studies) were excluded. Costs for studies done as part of program monitoring (including post distribution monitoring, PDM), and which could influence program implementation, were included.

All accounting databases provided by the humanitarian agency related to the project were analyzed to identify costs related to program implementation. Costs were included in the analysis once verified as relevant to the program activities by accounting and program staff. This included the cost of cash transfers, phones distributed to beneficiaries, staff training and monitoring. Costs were included primarily from the months of implementation, along with some months before and after where important implementation-related activities occurred (i.e. PDM or beneficiary follow-up).

Support costs were included in the analysis, including base office running costs, support staff (logistics, human resources, guards, cleaners), and equipment. Costs were separated into those relating to operations or research, and were allocated to the project according to total proportion of staff time dedicated to operational functions of the program.

Capital costs were extracted from the accounting data and amortized using standard tables (3 years for computers, 5 years for cars and other equipment). Costs of capital rental, such as cars, motorbikes, rooms and buildings, were classified as recurrent costs.

Implementation costs incurred by the private partner were estimated from a combination of summary budgetary data provided by partner accounting staff, and from private partner field staff accounts of resources used. These various data sources for the private partner’s expenditure data were cross-checked so that all program resources mentioned were included in the analysis. For example, any costs mentioned during interviews which were not included in the summary budgetary data were quantified using an ingredients approach to cost estimation, where estimates are constructed from unit costs and quantities of resource inputs [28]. Where there was no estimate available of the specific cost incurred for an item (e.g. a vehicle rental fee), estimates were used of costs for similar items from the humanitarian agency’s accounting records.

Data from community discussions with beneficiaries were analyzed in several ways. Both hand-written discussions and tape-recordings were translated into French by the note-taker. These transcripts were then translated into English by the French–English translator. Costs to beneficiaries reported in community discussions were analyzed for summary statistics (mean, median, max, min). Transcripts were analyzed for frequency of reported beneficiary livelihoods. To value beneficiaries’ time spent in the program, a daily shadow wage was used of 1000 CFA, based on the average daily income that women could earn from selling fritters, a common livelihood activity for women in the project area. Reported daily income from selling fritters ranged from 500 to 3500 CFA, the median was used to account for outliers.

Costs were converted into EUR each month in the project accountancy based on global exchange rates reported monthly from the humanitarian agency’s banking institution. Microsoft Excel software was used for analysis of cost data. For this analysis costs were converted to USD and adjusted for inflation using a GDP deflator. Results are presented in 2015 USD.

### Cost categorization

Costs were organized in multiple ways for the cost analysis.

#### Accounting categories

Expense data from different grant accounting databases were brought together and grouped according to common categories. These included personnel, program costs, and local office support costs. Total cost estimates include costs to implementing partners, community members and beneficiary households.

#### Input categories

The primary analysis of costs uses allocation to input categories as described in the DFID humanitarian budget format [29]. This includes the following subcategories: program inputs, including cash transfers; transport; security; overheads, including general logistics and field office costs; staffing and support; monitoring and evaluation (M&E); and capital items.

#### Activity-based costing

Key program activities were identified during data collection, and all costs allocated to these activity-based cost centers during analysis. Costs were allocated to activities based on direct usage where possible, and on estimates of staff time allocation to activities.

### Cost-efficiency

Cost-efficiency was estimated by calculating cost per program beneficiary and cost-transfer ratios. Cost per beneficiary and per household were calculated using the total program costs from a societal perspective, both including and excluding the value of the cash transfer. Cost-transfer ratios were calculated both from a societal and an institutional perspective, and both excluding the transfer costs (cost-transfer ratio, CTR) and including them (total cost-transfer ratio, TCTR).

Deterministic sensitivity analysis was conducted on the cost-efficiency results by varying several cost parameters one at a time while holding all other costs equal to the base case. Choice of parameters to vary in sensitivity analysis was made based on the input either comprising a relatively high proportion of overall costs (i.e. transport costs) or having potentially high uncertainty, including parameters from qualitative findings which may influence study outcomes (i.e. beneficiary wait time and shadow wage). Effects of these variations were calculated on the CTR and TCTR from both a societal and institutional perspective, as appropriate. The parameters assessed in the sensitivity analysis were:

- Transport costs were 25% cheaper, assuming staff could achieve marginal efficiencies in local transport.
- Assuming beneficiaries spent one-half day attending the distribution, rather than one full day as in the base case (and therefore reduced their opportunity costs by one-half).
- Beneficiary daily shadow wage was varied across a range of values cited in community discussions on livelihoods:
  - 1000 CFA (base case value): the median wage for selling fritters, a popular livelihood among women.
  - 500 CFA: the minimum wage for selling fritters.
  - 3500 CFA: the maximum wage for selling fritters.
  - 1250 CFA: the median for all livelihoods cited in community discussions.

### Qualitative analysis

Thematic analysis [30] was conducted by the primary researcher (CP). In Microsoft Word on electronic transcripts of interviews with staff from the humanitarian agency and private partner, and from community discussion with beneficiaries, according to themes related to implementation of electronic cash transfers identified in previous work [31], and to specific barriers faced by beneficiaries. The qualitative analysis further focused on identifying barriers to efficient implementation.

## Results

### Activity-based cost centers

Five key program activities were identified, as outlined in Table 2.

**Table 2:** Description of cost centers and data sources

Cost Center	Description	Data sources
1. Beneficiary identification	*Humanitarian agency* Time spent by staff before implementation began to select eligible beneficiaries in project area *Community members* Time spent as part of beneficiary identification committee	*Humanitarian agency* Review of financial documents. Time allocation interviews with staff. Discussion with staff on community member time allocation
2. Mobilization	*Humanitarian agency* Time spent by staff in monthly visits to villages informing beneficiaries of upcoming distributions, including related travel time	*Humanitarian agency* Review of financial documents. Time allocation interviews with staff
3. Distribution	*Humanitarian agency* Time spent by staff in monthly cash distribution. Program costs including cost of cash transfers, portable phones, and printing of beneficiary cards *Private partner* Time spent by staff in monthly cash distribution. Costs for security and vehicle rental. Overhead costs for fixed cash points *Beneficiaries* Time and transport costs in attending distributions	*Humanitarian agency* Review of financial documents. Time allocation interviews with staff *Private partner* Time allocation interviews with staff. Interviews regarding implementation costs *Beneficiaries* Community discussions regarding time and costs
4. Sensitization	*Humanitarian agency* Time spent by staff in awareness-raising at beginning of program, and at regular FGDs throughout implementation, along with related travel time *Beneficiaries* Time spent in monthly FGDs with humanitarian agency	*Humanitarian agency* Review of financial documents. Time allocation interviews with staff *Beneficiaries* Community discussions regarding time and costs
5. Follow-up and monitoring	*Humanitarian agency* Time spent staff in follow-up and monitoring after distribution. Program costs including post distribution monitoring (PDM) and mobile phone credit given to complaint management committee	*Humanitarian agency* Review of financial documents. Time allocation interviews with staff. Discussion with staff on community member time allocation

### Costs

Table 3 summarizes input costs per activity. The primary activity was the distribution itself at 74% of total costs. Other interactions with beneficiaries made up the remainder of the project activities, with mobilization (4%), sensitization (4%) and post-distribution follow-up and complaints management (10%) requiring a total of 18% of program resources. Identification of beneficiaries at the beginning of the program comprised 8% of program resources. The humanitarian agency incurred 89% of costs in the program, and estimates of private partner costs represent 5% of total implementation costs. For societal costs, household costs represent 6% of total program costs, and community costs represent less than 1% of total program costs.

**Table 3:** Input costs per activity—MAM’Out

Cost center inputs	Costs (USD)	% of total
Beneficiary identification	28,006	7.9
Personnel	13,302	3.8
Community costs		
Time of beneficiary identification committee	490	0.1
Support costs allocated	14,214	4.0
Mobilization	13,816	3.9
Personnel	5519	1.6
Support costs allocated	8297	2.3
Distribution	262,735	74.3
Personnel	5519	1.6
Program costs		
Cash transfers	194,640	55.0
Portable phones	16,553	4.7
Printing beneficiary cards	92	0.0
Partner costs		
Mobile cash points		
Partner staff	7959	2.3
Security: gendarme	2608	0.7
Vehicle rental	6013	1.7
Fixed cash points		
Distributor staff	249	0.1
Security: guards	132	0.0
Building overhead	203	0.1
Beneficiary costs		
Time at distribution (waiting, travel + participation)	17,343	4.9
Transport to/from distribution	3127	0.9
Support costs allocated	8297	2.3
Sensitization	15,459	4.4
Personnel	5523	1.6
Beneficiary costs		
Time participating in sensitization	854	0.2
Support costs allocated	9082	2.6
Follow-up and monitoring	33,684	9.5
Personnel	10,838	3.1
Program costs		
Phone credit for Complaints Management Committee	4348	1.2
Monitoring of markets and food situation	377	0.1
Post distribution monitoring	1219	0.3
Community costs		
Time of Complaints Management Committee	608	0.2
Support costs allocated	16,295	4.6
Total societal costs	353,700 USD	
Institutional costs	331,278	93.7
Humanitarian agency costs	314,114	88.9
Private partner costs	17,164	4.9
Community costs	1098	0.3
Beneficiary costs	21,325	6.0

**Fig. 1:**
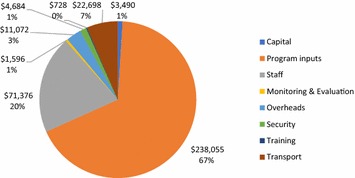
Input costs

Figure 1 presents costs by input category. Staff costs made up 20% of program costs. Transport was 7% of the total. The following input categories were all 3% or less of total program costs: capital, monitoring and evaluation costs, overhead, security, and training. Specific program inputs were 67% of all costs, 82% of this was the cost of the transfer. Mobile phone costs were 9% of these program input costs, including the cost of portable phones given to beneficiaries and cost of airtime given to members of the complaint management committee as reimbursement for their time. Beneficiary costs made up 9% of program input costs, including the value of time spent at distribution and community discussions, and direct costs for transport to the distribution. Costs to community members in identifying beneficiaries and participating in the complaints management committee were 0.5% of input costs. The cash transfer itself was over half of total program costs (55%), compared to all other costs included in the analysis (45%). Program costs were comprised of 1% fixed capital costs and 99% variable recurrent costs.

**Fig. 2:**
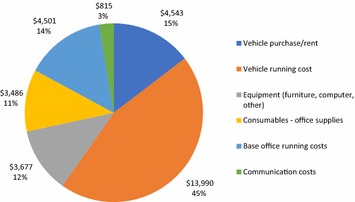
Local office costs

For costs related to supporting the local office, as shown in Fig. 2, vehicles and running costs were over 60% of costs in this category, partly due to the project area being spread out and staff traveling frequently, often to remote areas, for the project activities.

**Fig. 3:**
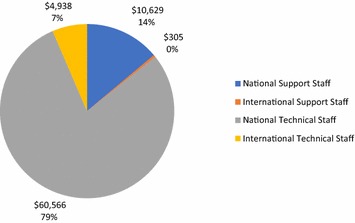
Staff costs

Personnel costs were 22% of total costs. The staff profile was primarily national staff, at 93% of total staff costs as shown in Fig. 3.

**Fig. 4:**
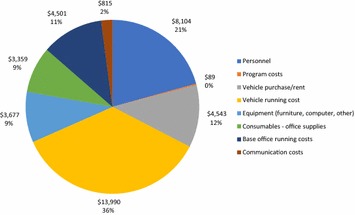
Support costs

Support costs made up 11% of total costs. As shown in Fig. 4, the cost of vehicles, including rental (12%) and running costs (36%) together made up the largest cost shares for support costs, at 48% of total support costs incurred in the program. Support personnel made up another 21% of costs. Equipment (9%), Office supplies (9%) and base office running costs (11%) each made up approximately 10% of the support cost category. Communication costs (2%) and translator support hired for communicating with some villages (< 1%) made up the remainder of costs in this category.

**Fig. 5:**
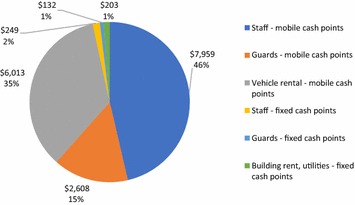
Private partner costs

Figure 5 presents costs to the private partner. Their primary costs were the staff employed to oversee mobile cash points (46%), followed by rental of vehicle for mobile cash points (35%) and guards employed for mobile cash points (15%). For fixed cash points, 2% of total costs went to staff overseeing these fixed points, 1% of costs went to guards overseeing the fixed points, and 1% of costs went to the overheads for the buildings used for fixed points. Overall, nearly 96% of private partner costs were dedicated to operating the mobile cash points, and 4% of costs went to operating fixed cash points during cash distributions for MAM’Out.

The majority of resources used by households to participate in the program (81%) went to the value of the time spent by women at the distribution site itself, most of which was time spent waiting for the distribution team to arrive, particularly at the mobile sites (range = 15 min to 8 h). Transport to the distribution represented 15% of household costs. Time spent by beneficiaries at the monthly FGDs in their communities during the cash transfer periods represented 4% of the total resources required from beneficiary households to participate in the project.

### Cost efficiency

Cost-efficiency results are summarized in Table 4, estimated both from a societal perspective, including costs to communities and households, as well as from an institutional perspective, including only costs to the humanitarian agency and the private partner.

**Table 4:** Cost-efficiency results—MAM’Out

	Results
Total societal costs^a^	353,700
Total institutional costs	331,278
Outputs	
Number of beneficiary households	856
Number of children in program	900
Cost per household, societal perspective, including transfer value	413
Cost per household, societal perspective, excluding transfer value	186
Cost per beneficiary, societal perspective, including transfer value	393
Cost per beneficiary, societal perspective, excluding transfer value	177
Cost-transfer ratio^b^, societal perspective	0.82
Total cost-transfer ratio^c^, societal perspective	1.82
Cost-transfer ratio^b^, institutional perspective	0.70
Total cost-transfer ratio^c^, institutional perspective	1.70

^a^All costs are presented in 2015 USD

^b^The cost to transfer one dollar to a beneficiary, excluding the transfer costs

^c^The total cost to transfer one monetary unit to a beneficiary, including the value of the transfer itself

The cost transfer ratios can be interpreted to mean that it cost the program 70 cents to deliver every dollar to beneficiaries; when including costs borne by communities and households, this figure increases to 82 cents spent for every dollar delivered.

Results from the sensitivity analysis on cost-efficiency outcomes are presented in Table 5. These results demonstrate that adjusting uncertain parameters across a range of plausible values did not result in a large change in cost-transfer ratio or total cost-transfer ratio, from an institutional or societal perspective. The largest change observed was when assuming a reduced wait time for beneficiaries at mobile cash points; however, at most this resulted in a reduction of the societal CTR by 6%.

**Table 5:** Cost efficiency sensitivity analysis

Adjusted parameter	Cost-transfer ratio		Total cost-transfer ratio	
	Institutional	Societal	Institutional	Societal
Base case	0.70	0.82	1.70	1.82
Transport costs	0.68	0.80	1.68	1.80
Beneficiary wait time	–	0.77	–	1.77
Daily wages				
Median of all livelihoods	–	0.82	–	1.82
Women’s livelihood^a^—maximum wage	–	0.83	–	1.83
Women’s livelihood^a^—minimum wage	–	0.81	–	1.81

^a^Selling fritters was a common livelihood for women in the project area; the median daily wage for selling fritters was used in the base case analysis

### Qualitative findings

Several themes were identified during qualitative thematic analysis of the interviews and community discussions, related to common issues in implementation of electronic cash transfers, namely private sector partnership, security, and accessibility to recipient.

#### Private sector partnership

A primary theme arising from interviews was the relationship between the humanitarian agency and the private mobile phone company assisting with the mobile money program.

Interviewees had different perceptions of the cause of inefficiencies in the distribution implementation, particularly in the first year of the program.

Humanitarian agency staff observed that the private sector partner had to travel 230 km to their regional base which was not accounted for in planning. They would settle a time for women to arrive for the distribution at 8 AM. “[The private partner staff] leave Koupela, get the money in Fada, and it would start at 10 AM. Some [women] can’t wait that long. This was a challenge in the first year only.”

—Humanitarian agency staff

“Sometimes people would have to wait because [the private partner] didn’t refill their accounts so there was not enough money to pay all the beneficiaries.”

—Bank teller (at private cash point offering mobile money services)

“The principle challenge was to respect the timeline set by [the humanitarian agency] on distribution days. At beginning, it was difficult to combine those schedules… The issue wasn’t with us, we already offered this service to other clients. More the staff at [the humanitarian agency], to know how the distribution could be more efficient.”

—Private partner staff

“The coordination went well, what to improve is at [our] level, to improve the number of cash points, increase availability of cash points in the area. This is not [the humanitarian agency’s] role.”

—Private partner upper management

“In the future, to increase effectiveness [the humanitarian agency] should recruit an agent who knows more about telepayment, someone who is well-informed of cash transfer by mobile phone. This person would be in charge of organizing the various partners. At [our] level, we should extend coverage of cash points to give more availability to beneficiaries. The cash transfer is an effective tool because in these areas, the security is a concern. Imagine if [the humanitarian agency] staff must go to these villages with cash on them. For me, it’s a great tool, if we have a cash point in the village, a beneficiary can go get cash at her own convenience, she doesn’t need to be there at a set time.”

—Private partner staff

Mobile cash points were expensive for the private partner to implement and provided little profit. Bank tellers of branches in Kantchari town who offered mobile money services and were involved in the distribution estimated that the cash transfer represented between 5 and 10% of their total business on the distribution days. Some mentioned that the profitability was acceptable because the transfer was done every month.

The cost of operating the mobile cash points influenced implementation in multiple ways and gave rise to attitudinal barriers, and distrust from the NGO partner about the underlying motives of the private partner.

“The mobile cash point, it is not necessarily in a building… We move to the people, for two reasons: one, the distance outlined in the agreement, and two, there is no benefit for [us] to make a new cash point in the area.”

—Private partner staff

“In Tapoa we have only 15 cash points. One of the goals for this project is to bring money closer to the beneficiaries. But we know it’s difficult to do this. We can’t just put a cash point there for beneficiaries, we look for a good yield. We assess, where is there a good medium, close enough to beneficiaries but not too remote.”

—Private partner staff

Humanitarian agency staff mentioned that the private partner wanted to save money by not committing a big team for each distribution, “because they’re capitalists!”

—Humanitarian agency staff

“If you have to analyze the resources dedicated to activities, [we don’t] earn much.”

—Private partner staff

Despite lack of profitability for the private partner, they did also see some benefits to the project.

“It would be good to continue the collaboration. It’s something good for the village. People recognize us. When I go to different villages to visit friends, others see me and say “You’re the [private partner] people who give us money!” If we stop [the distributions] then the people wouldn’t be happy. It also widens [the private partner’s] relationships, with these different beneficiaries and with [the humanitarian agency].”

—Bank teller (at private cash point offering mobile money services)

#### Security

There were security issues, including banditry on roads, in the rural areas where the intervention took place. Due to heightened concern around transporting cash in this environment, investments were made by the private partner in gendarmes and unmarked cars for distributions. This detracted from the private partner’s profit.

Managers at the humanitarian agency mentioned that they asked the private partner to commit more staff to distributions to improve speed and efficiency, but the private partner said security was a concern, and wanted to have fewer staff involved. From the private partner side, the morning of the distribution they would need to pick up the gendarmes, go to the central office to pick up the cash, and then pick up other staff involved in the distribution. All of this coordination frequently resulted in delays against the planned timeline.

The humanitarian agency and the private partner kept in contact in the days leading up to the distribution to discuss any security events and the possible need to change location and dates of distribution.

“Not all the trips to mobile points need security, only where the risk of insecurity is higher. We never faced insecurity in the village, more on the road. In the village, we have the community around us. Two cash points out of ten needed security. For fixed cash points, we didn’t need to bring cash from anywhere. For mobile points, we needed to bring in mobile cash points or have another partner who moves the cash there.”

—Private partner staff

“We will always have one gendarme in the front seat and one in the back seat. If we go to a village where we need less staff, one car is enough. Otherwise, we need more staff, two cars and two more gendarmes.”

—Private partner staff

#### Accessibility to recipient

Delays in the project implementation during some distribution periods had important effects on the accessibility of the program for beneficiaries.

“Because of waiting so long, some women can’t cook dinner when they get back home, it is too late to cook.”

—Focus group discussion #2 with beneficiary women, reporting a 6 h wait at distribution site

“Sometimes, we have to come back the next day.”

—Focus group discussion #4 with beneficiary women, reporting a 5 h wait at distribution site

“For all the ladies, the waiting time is too long.”

—Focus group discussion #5 with beneficiary women, reporting an 8 h wait at distribution site

“[Women wait for] seven hours max, also sometimes [the private partner] didn’t arrive. [The private partner] can do three villages in one day, this makes women wait. Normally women wait 4-5 h, and the cash distribution takes 4-5 h. This depends also on the number of beneficiaries [attending each distribution] and how the network functions.”

—Humanitarian agency staff

“My perception is that 10,000 CFA is not enough and there are women who are very far. When they cancel [the distribution], they have to sleep here. Two women slept for two nights in Kantchari. [Usually] they will spend the whole day to travel, and come wait. They won’t work on their other activities, just come to the cash points… There is no wait at fixed cash points, it goes smoothly. No one would know that you were there to get money.”

—Humanitarian agency staff

## Discussion

This study presents results on the costs and cost-efficiency of a mobile cash transfer distributed during the lean season to prevent child undernutrition in rural Burkina Faso. The cost-efficiency of the program, with a cost-transfer ratio of 0.82 when including costs to beneficiary communities and 0.70 including only costs to implementing institutions, was found to be within the same range as other similar interventions. However qualitative findings from interviews with staff from the humanitarian agency and the private partner organization providing the mobile money services indicate that the efficiency of implementation could have been improved with more time to fine tune coordination beyond this relatively short-term pilot study experience. This finding was reinforced by another qualitative analysis of this trial which found an improvement in coordination in the second year of the cash transfer [15].

The TCTR for mobile transfers in this setting was 1.82, from a societal perspective and including the cost of the transfer itself; this figure is 1.70 including only costs to implementing institutions. These results appear to be in the same range as other humanitarian transfer programs. A review of ECHO-funded transfer programs found that 75% of these achieved a TCTR of less than 2 across different transfer modalities [6]. Values can range widely around this average, from a TCTR of 1.11 for a cash transfer in Yemen in 2013 [8], to an average TCTR of 2.81 for 27 cash programs implemented in complex emergencies [6]. The cost-transfer ratio for the MAM’Out intervention was 0.70 from an institutional perspective. Other electronic transfers specifically have reported CTRs—including only institutional costs—on the lower side of the above-cited range, for example 0.15 to deliver food and cash, and 0.29 to deliver cash alone in two programs in Kenya, and 0.20 for UCTs in Somalia [32]. This supports findings from other settings [33] suggesting that mobile transfers can provide a more cost-efficient alternative to other cash transfer modalities, due to, for example, a reduction in costs of implementation associated with manual distribution of cash, and a decrease in leakage by transferring money directly to recipients. The differences in CTRs indicate that the MAM’Out intervention did not achieve the same degree of cost-efficiency as has been achieved by other mobile transfer programs. This could be explained in part by this being the first time the humanitarian agency had used mobile phones to transfer money in this area; qualitative data indicates that improvements in coordination were seen in the second year of the program once systems were better-established.

The cost per child beneficiary in this program was 393 USD from a societal perspective including the transfer, and 177 USD excluding the transfer cost. This cost is comparable to other values reported for cash transfer programs which include costs to beneficiaries, including those cited in a recent systematic review by Doocy and Tappis [8] with costs per beneficiary of 175 USD in Yemen, and other recent estimates of 127 USD per beneficiary in Niger [34]. Comparison of results is challenged by differing contexts, and also because many cost-efficiency studies do not provide a thorough and transparent documentation of which costs are included or excluded, and therefore are potentially incomparable with those from the present study. Efforts should be made to improve transparency in reporting of future costing studies.

One objective of using cash for nutrition outcomes is to achieve more efficiency and greater freedom in use of the transfer than is allowed when distributing food directly as nutrition-specific interventions do. Community management of acute malnutrition programs regularly achieve costs per beneficiary of between 135 USD and 200 USD, with the lipid nutrient supplements (LNS) comprising between 24 and 51% of total costs in recent studies [24, 35–37]. While these programs have similar target populations to the present study and also address undernutrition, their objective is to provide therapeutic treatment which carries a substantively different cost structure with intervention resources focused on individuals rather than the preventive intervention addressed here, which intervenes at a population level; therefore direct comparisons of program costs are discouraged. An intervention in Chad providing a LNS alongside a distribution of staple rations to prevent child undernutrition incurred a cost per beneficiary of 728 EUR for the staple ration alone (approximately 990 in 2017 USD), and an additional 374 EUR (508 in 2017 USD) for each child receiving the LNS [25]. The staple ration made up 49% of total costs, LNS comprised less than 1% of costs due to the small number of children covered in this study setting; these results are similar to those in the MAM’Out trial in that distributed resources also comprised approximately one-half of program costs, though cost per beneficiary in the present study was significantly less. This intervention did not achieve a significant effect in reducing the incidence of undernutrition among children, nor did the MAM’Out intervention.

Transport costs were high in this setting, since the large and sparsely populated intervention area required that staff travel frequently to remote villages. The monthly distribution cycle necessitated several staff visits to each village, for mobilization before the distribution which included a discreet announcement of the next distribution date to avoid security concerns, a visit for the distribution itself, and a visit for post distribution follow-up and monitoring. Implementing this mobile transfer in a more accessible area may have increased efficiency, however in sensitivity analyses, assuming improved efficiency in transport costs did not result in a substantive change in the CTR.

Beyond the possible marginal reductions to be made in transport costs, a more important adjustment to the intervention’s efficiency might have been in enhancing coordination with the private partner to improve speed and efficiency of the monthly distributions themselves. This problem was raised in community discussions, and there were some cases of beneficiaries waiting days to receive their transfer, particularly when attending mobile cash points. Modeling a reduced wait time in sensitivity analyses resulted in a marginal improvement of 6% reduction in CTR; this was the largest change seen of any study parameters investigated in the sensitivity analysis. Aker et al. [33] found that compared to manual cash transfers, mobile transfers in Niger reduced the opportunity cost of beneficiaries. The inefficiencies and coordination challenges experienced in the present study setting are likely due to this having been a pilot project; as is common with mobile transfers, cost efficiencies commonly are realized over time and with improved systems and network functioning [32].

The qualitative analysis uncovered several barriers in partners’ experience of implementing the program, all of which are common for mobile transfer interventions [31]. These included financial barriers experienced by the private partner in expanding their services into remote areas, especially those requiring investments for security. Both political and attitudinal barriers resulted from the various partners’ suspicions of one another’s motives. In the case of the humanitarian agency, there was some distrust of the private partner’s profit motive. For the private partner, there was a perception of the humanitarian agency as being unfamiliar with mobile money and unable to manage such a program efficiently. These challenges were perceived as being stronger at the beginning of the program and before a more trusting relationship and efficient system were established. These findings again point to the potential for mobile transfers to gain efficiencies as processes are standardized and experience gained over time, and suggest that trusting relationships between partners, and preferably an established prior relationship working together on mobile money, are important factors supporting efficiency.

This study has several limitations. First, we did not have access to partners’ accounting systems, and so estimates of partner costs were collected via interview and from summary budgetary information provided by partner accounting staff, therefore some resources may not have been captured leading to an underestimation of costs. However questions were asked during interviews about all areas of anticipated costs. Second, beneficiary cost data is based on qualitative discussions and may not be statistically representative of the entire study population. However FGD sites were purposively selected to represent a range of geographic locations in the project area and to include beneficiaries from mobile and fixed sites; further the qualitative nature of the discussions enabled a good understanding of participants’ experiences in the intervention. We were not able to assess cost-effectiveness of the mobile transfer intervention due to there being no significant impact on the primary outcome of acute malnutrition incidence [13]; though impacts were found on secondary outcomes [14]. Finally, mobile transfers have a higher chance of being cost-efficient in longer-term programs with well-functioning systems in place and with amortization of startup costs for the technologies used [32], therefore findings from this pilot study may underestimate the cost-efficiency of the intervention and not be generalizable to other settings with more well-established mobile money networks.

Despite these limitations, this study has filled a gap in the evidence base by providing a detailed and rigorous analysis of costs to implementing agencies and beneficiaries, and cost-efficiency of a mobile cash transfer implemented to address child undernutrition, complemented by qualitative analysis of barriers encountered by implementing partners and beneficiaries, to inform future program implementation. If local government stakeholders were interested to implement such a program, it is likely that costs would be reduced greatly compared to this study context. Specifically, implementation by government might improve cost-efficiency if the government could subsidize expansion of mobile money network coverage and increase the number of cash distribution points in remote areas such as those in the present study in Tapoa province, which would not be profitable for private partners. Further, having the government as the sole implementer could improve efficiency and reduce costs, for example for transport and salaries, though this would depend on other factors. Finally, it is possible that government might choose a different implementation modality than mobile accounts. For example, savings accounts might instead be used from which beneficiaries could withdraw money at their convenience. Government also might choose a “virtual money” approach using smart cards or e-vouchers to create a chain of cashless payments for goods and services; these approaches usually require investments from the state to reach adequate scale and establish a wide network of participating vendors. Such approaches, while common in East Africa, currently have limited reach in West Africa and offer great potential for addressing security concerns typically associated with cash programming.

## Conclusion

This study presents results from a mixed methods analysis of costs and cost efficiency of a mobile transfer program implemented to address child undernutrition in rural Burkina Faso. While the mobile transfer program achieved a cost-efficiency within the same range as other humanitarian transfer programs, it required a long wait for beneficiary households on distribution days, particularly at mobile cash points and in the first year of implementation. This inefficiency was due primarily to security challenges and coordination issues between the humanitarian agency and private partner implementing the mobile money activities. Actors reported that coordination issues improved over the course of the project, therefore these inefficiencies likely would be resolved over time, and cost-efficiency therefore improved, as the program moved past the pilot phase. Despite the time required to establish trusting relationships and functioning systems among actors, and to set up a network of cash points in remote areas, this analysis showed that mobile transfers hold promise as a cost-efficient method of delivering cash in this setting. Further work should be undertaken in rural Burkina Faso, by government with support from private partners and humanitarian stakeholders, to establish networks and expand mobile cash points to pave the way for more efficient future mobile transfer programming in this context.

## References

[1] Glassman A, Duran D, Fleisher L, Singer D, Sturke R, Angeles G, Charles J, Emrey B, Gleason J, Mwebsa W, Saldana K (2013). Impact of conditional cash transfers on maternal and newborn health. J Health Popul Nutr.

[2] Grellety E, Babakazo P, Bangana A, Mwamba G, Lezama I, Zagre NM, Ategbo EA (2017). Effects of unconditional cash transfers on the outcome of treatment for severe acute malnutrition (SAM): a cluster-randomised trial in the Democratic Republic of the Congo. BMC Med.

[3] Gaarder MM, Glassman A, Todd JE (2010). Conditional cash transfers and health: unpacking the causal chain. J Dev Eff.

[4] Manley J, Gitter S, Slavchevska V (2012). How effective are cash transfer programmes at improving nutritional status? A rapid evidence assessment of programmes’ effects on anthropometric outcomes.

[5] Buston O, Smith K (2013). Global humanitarian assistance report 2013.

[6] Maunder N, Dillon N, Smith G, Truelove S (2015). Evaluation of the use of different transfer modalities in ECHO humanitarian aid actions 2011–2014.

[7] ODI (2015). Doing cash differently: how cash transfers can transform humanitarian aid.

[8] Doocy S, Tappis H (2016). Cash-based approaches in humanitarian emergencies: a systematic review.

[9] Hodges A, White P, Greenslade M (2011). Guidance for DFID country offices on measuring and maximising value for money in cash transfer programmes.

[10] Hidrobo M, Hoddinott J, Peterman A, Margolies A, Moreira V (2014). Cash, food, or vouchers? Evidence from a randomized experiment in northern Ecuador. J Dev Econ.

[11] Hoddinott J, Sandström S, Upton J (2014). The impact of cash and food transfers: evidence from a randomized intervention in Niger.

[12] Tonguet-Papucci A, Huybregts L, Aït-Aïssa M, Huneau JF, Kolsteren P (2015). The MAM’Out project: a randomized controlled trial to assess multiannual and seasonal cash transfers for the prevention of acute malnutrition in children under 36 months in Burkina Faso. BMC Public Health..

[13] Houngbé F, Tonguet-Papucci A, Altare C, Aït-Aïssa M, Huneau JF, Huybregts L, Kolsteren P (2017). Unconditional cash transfers do not prevent children’s undernutrition in the moderate acute malnutrition out (MAM’Out) cluster-randomized controlled trial in rural Burkina Faso. J Nutr..

[14] Tonguet-Papucci A, Houngbe F, Huybregts L, Ait-Aissa M, Altare C, Kolsteren P, Huneau JF (2017). Unconditional seasonal cash transfer increases intake of high-nutritional-value foods in young burkinabe children: results of 24-hour dietary recall surveys within the moderate acute malnutrition out (MAM’Out) randomized controlled trial. J Nutr.

[15] Tonguet-Papucci A, Houngbé F, Lompo P, Yameogo WM, Huneau JF, Aït-Aïssa M, Kolsteren P (2017). Beneficiaries’ perceptions and reported use of unconditional cash transfers intended to prevent acute malnutrition in children in poor rural communities in Burkina Faso: qualitative results from the MAM’Out randomized controlled trial. BMC Public Health..

[16] United Nations Development Program (2016). Human development report 2016: human development for everyone: brief note for countries on the 2016 human development report—Burkina Faso.

[17] Ministry of Social Action and National Solidarity (2012). Social protection national policy 2013–2022.

[18] UN Office for the Coordination of Humanitarian Affairs. Overview of humanitarian needs 2016: Burkina Faso; 2015.

[19] Ouedraogo-Nikiéma L (2012). Evaluation de la situation nutritionnelle des enfants de 6 à 59 mois dans le district sanitaire de Diapaga: Enquête SMART du 25 avril au 10 mai 2012.

[20] Boucher-Castel L, Chalimbaud J (2013). Report NCA-Tapoa, Burkina Faso.

[21] Bargo M, Dahani M, Gandema A, Hien S, Konate PS, Ouedraogo D (2011). Household economic approach: southeast area in Burkina Faso, cereals, livestock farming, forest and fauna in Tapoa province: November–December 2011.

[22] Samson M, Cherrier C (2009). Etude de faisabilité pour un programme de transferts sociaux monétaires comme instrument majeur d’une protection sociale centrée sur l’enfant au Sénégal.

[23] National Institute for Statistics and Demography (2015). Continuing multi-sectorial studies 2014 poverty and inequalities profiles.

[24] Puett C, Sadler K, Alderman H, Coates J, Fiedler JL, Myatt M (2013). Cost-effectiveness of the community-based management of severe acute malnutrition by community health workers in southern Bangladesh. Health Policy Plan.

[25] Puett C, Salpéteur C, Lacroix E, Houngbé F, Aït-Aïssa M, Israël AD (2013). Protecting child health and nutrition status with ready-to-use food in addition to food assistance in urban Chad: a cost-effectiveness analysis. Cost Eff Resour Alloc.

[26] Puett C, Salpéteur C, Lacroix E, Zimunya SD, Israël AD, Aït-Aïssa M (2014). Cost-effectiveness of community vegetable gardens for people living with HIV in Zimbabwe. Cost Eff Resour Alloc.

[27] Puett C. Assessing cost-effectiveness of interventions within a humanitarian organization. Disasters. 2018 (**in press**). 10.1111/disa.12344 PMC6850649 31012136

[28] Edejer TT (2003). Making choices in health: WHO guide to cost-effectiveness analysis.

[29] Department for International Development (2015). DFID humanitarian response funding guidelines for NGOs.

[30] Saldaña J (2015). The coding manual for qualitative researchers.

[31] Smith G, MacAuslan I, Butters S, Tromme M (2011). ‘New technology enhancing humanitarian cash and voucher programming’, a research report commissioned by CaLP.

[32] O’Brien C, Hove F, Smith G (2013). Factors affecting the cost-efficiency of electronic transfers in humanitarian programmes.

[33] Aker J, Boumnijel R, McClelland A, Tierney N (2013). How do electronic transfers compare? Evidence from a mobile money cash transfer experiment in Niger.

[34] Trenouth L (2017). REFANI synthesis report.

[35] Bachmann MO (2009). Cost effectiveness of community-based therapeutic care for children with severe acute malnutrition in Zambia: decision tree model. Cost Eff Resour Alloc.

[36] Wilford R, Golden K, Walker DG (2012). Cost-effectiveness of community-based management of acute malnutrition in Malawi. Health Policy Plan.

[37] Tekeste A, Wondafrash M, Azene G, Deribe K (2012). Cost effectiveness of community-based and in-patient therapeutic feeding programs to treat severe acute malnutrition in Ethiopia. Cost Eff Resour Alloc.

